# Molecular Basis of Antigenic Drift in Serotype O Foot-and-Mouth Disease Viruses (2013–2018) from Southeast Asia

**DOI:** 10.3390/v13091886

**Published:** 2021-09-21

**Authors:** Sasmita Upadhyaya, Mana Mahapatra, Valerie Mioulet, Satya Parida

**Affiliations:** 1 The Pirbright Institute, Ash Road, Pirbright GU24 ONF, UK; sasmita.upadhyaya@yahoo.com (S.U.); mana.mahapatra@pirbright.ac.uk (M.M.); valerie.mioulet@pirbright.ac.uk (V.M.); 2 Food and Agriculture Organization of the United Nations (FAO), 00153 Rome, Italy

**Keywords:** Southeast Asia, East Asia, Foot and Mouth Disease (FMD), serotype O, vaccine strain selection

## Abstract

Foot and mouth disease (FMD) is a highly contagious disease of cloven-hoofed animals with serious economic consequences. FMD is endemic in Southeast Asia (SEA) and East Asia (EA) with the circulation of multiple serotypes, posing a threat to Australia and other FMD-free countries. Although vaccination is one of the most important control measures to prevent FMD outbreaks, the available vaccines may not be able to provide enough cross-protection against the FMD viruses (FMDVs) circulating in these countries due to the incursion of new lineages and sub-lineages as experienced in South Korea during 2010, a FMD-free country, when a new lineage of serotype O FMDV (Mya-98) spread to the country, resulting in devastating economic consequences. In this study, a total of 62 serotype O (2013–2018) viruses selected from SEA and EA countries were antigenically characterized by virus neutralization tests using three existing (O/HKN/6/83, O/IND/R2/75 and O/PanAsia-2) and one putative (O/MYA/2009) vaccine strains and full capsid sequencing. The Capsid sequence analysis revealed three topotypes, Cathay, SEA and Middle East-South Asia (ME-SA) of FMDVs circulating in the region. The vaccines used in this study showed a good match with the SEA and ME-SA viruses. However, none of the recently circulating Cathay topotype viruses were protected by any of the vaccine strains, including the existing Cathay topotype vaccine (O/HKN/6/83), indicating an antigenic drift and, also the urgency to monitor this topotype in the region and develop a new vaccine strain if necessary, although currently the presence of this topotype is mainly restricted to China, Hong Kong, Taiwan and Vietnam. Further, the capsid sequences of these viruses were analyzed that identified several capsid amino acid substitutions involving neutralizing antigenic sites 1, 2 and 5, which either individually or together could underpin the observed antigenic drift.

## 1. Introduction

Foot and mouth disease (FMD) is an economically devastating and highly contagious vesicular disease affecting domestic and wild cloven-hoofed animals [1,2,3]. The disease has been detected in more than 100 countries worldwide; most of Asia including Southeast Asia (SEA) and East Asia (EA), Africa and the Middle East are FMD-endemic, whereas Australia, North America, Western Europe and Japan are free from FMD. In South America, FMD is controlled by vaccination. Generally, FMD shows high morbidity and low mortality in adult animals; however, mortality could be very high in young animals (50–80% in calves and up to 100% in piglets) because of myocarditis. The disease can cause a significant production loss in infected animals and results in trade barriers causing serious damage to the economy of countries by restricting the exports of livestock and livestock products.

The causative agent, FMD virus (FMDV), is a non-enveloped single-stranded, positive-sense RNA virus, belonging to the Family *Picornaviridae* in genus *Aphthovirus*. The viral capsid is formed by four viral structural proteins (VP1–VP4); VP1, VP2 and VP3 are surface-exposed and contain most of neutralizing antigenic sites, whereas VP4 is internal [4]. Due to the lack of the proof-reading activity of the RNA polymerase and the quasispecies nature of the virus, there is a greater chance of evolving new variants of FMDV, resulting in the existence of seven immunologically distinct serotypes of FMDV O, A, C, Asia 1, South African territories (SATs) 1–3, and many subtypes within a serotype. Of these, serotype C has not been detected since 2005 and is thought to be extinct [5]. Among the six serotypes, serotype O is the most predominant worldwide followed by serotype A [1,2,3]. Vaccination is one of the most important measures to control FMD; however, the antigenic variation creates a major problem to control FMD outbreaks by using conventional inactivated virus vaccines, as vaccination against one serotype of FMDV does not provide cross-protection against other serotypes and, also sometimes does not provide complete protection against other subtypes within the same serotype [2,3]. Therefore, it is of utmost importance to conduct regular vaccine-matching tests between available bovine post-vaccinal sera (BVS) and circulating viruses, and if necessary, new vaccine strains need to be selected for providing better protection [6].

FMD is endemic in Asia, except for Japan. Three serotypes of FMDV (O, A and Asia 1) are usually circulating in SEA and EA countries. Most of the FMD outbreaks in the regions are caused by serotype O followed by serotype A [7,8]. There have been reports of continuous FMD outbreaks since 2010–2015 in different countries in SEA, Far East and East Asia (such as South Korea, Vietnam, Lao and Thailand) [9,10,11]. Since 2010, a new strain of serotype O, Myanmar-98 (Mya-98), have been circulating in many countries in SEA, Far East and EA. Along with three usual strains O/SEA/Mya-98, O/Middle East-South Asia (ME-SA)/PanAsia and Cathay, a new O/ME-SA/Ind-2001 lineage, has been circulating in this region since 2015 and, have now spread to most of the SEA countries (Table 1). Although vaccination is the key to controlling FMD, the available vaccines are not able to provide enough cross-protection as outbreaks have been still seen in South Korea since 2010 even with repeated vaccinations [12,13,14]. Therefore, a regular vaccine-matching test of circulating viruses with the existing vaccines in antigenic reserves is required, and if necessary, a broadly cross-reacting vaccine strain should be used in vaccination programs. Previously, we reported that four out of the five vaccine strains (*v*/*s*) that were tested in in vitro vaccine-matching tests exhibited relatively higher cross-reactivity (84–95%) with serotype O FMDVs circulating in SEA countries during 2007–2012. In this paper, we report the antigenic and genetic characterization of serotype O viruses isolated from SEA countries between 2013 and 2018. The overall cross-reactivity of the BVS was found to be relatively lower (63–73%), indicating the alteration in the antigenicity of the serotype O viruses from the region, especially the Cathay viruses from Hong Kong.

## 2. Materials and Methods

### 2.1. Cells, Viruses and BVS

A total of 62 serotype O FMDVs submitted to the World Reference Laboratory for FMD (WRLFMD) at Pirbright were selected for this study (Table S1). These viruses were isolated over a six-year period between 2013 and 2018 from nine SEA and EA countries, namely Cambodia (*n* = 5), Hong Kong (*n* = 17), Lao (*n* = 3), Malaysia (*n* = 6), Mongolia (*n* = 3), Myanmar (*n* = 1), South Korea (*n* = 3), Thailand (*n* = 8) and Vietnam (*n* = 16) (Table 2a). These samples were collected from either cattle (*n* = 32), pig (*n* = 27) or water buffalo (*n* = 2) epithelial tissues, except two viruses from Lao, of which the host species was not known (Table S1). In addition, four vaccine/homologous viruses (O/PanAsia-2, O/MYA/2009, O/HKN/1983 and O/IND/R2/75) were included for vaccine-matching studies, making it a total of 66 viruses. The history of these vaccine viruses has been described previously [12,15,16]. All these viruses were grown initially in primary bovine thyroid (BTY) cells with a subsequent passage (two to three times) in IBRS2 cells (pig kidney cell line). The virus stocks were prepared by infecting IBRS2 cell monolayers with ~90% confluency that were incubated in a 37 °C incubator with 5% CO_2_. When a more than 85% cytopathic effect (CPE) was observed, cell culture supernatant fluids containing virus were clarified by centrifugation at 2500 rpm for 15 min. Following harvest, the virus suspension was stored at −70 °C as single-use aliquots. The IBRS2 cells were also used for virus titration and serological assays.

BVS: Four BVS, namely O/PanAsia-2 (ME-SA/PanAsia-2 strain), O/IND/R2/75 (ME-SA topotype), O/HKN/1983 (Cathay topotype) and O/MYA/2009 (SEA/Mya-98 strain), were used in this study and have been described previously [12,15,16]. Of these BVS, O/IND/R2/75, O/PanAsia-2 and O/HKN/1983 were against existing *v*/*s*, while O/MYA/2009 [12] was raised in bovine against a putative strain. For each vaccine antigen, a pool of sera from five animals was used in the serological tests.

### 2.2. Virus Ttitration and Two-Dimensional Micro-Neutralisation Test (2D-VNT)

Virus titration was carried out on IBRS2 cells at least twice to obtain the mean virus titre and expressed as a 50% log_10_ tissue culture infectious dose (TCID)_50_/mL. Viruses with a titre of 3.5 log_10_ TCID_50_/mL or greater were used for the 2D-VNT. The 2D-VNTs were conducted using pooled bovine sera according to the method described by Rweyemamu and colleagues [17]. Antibody titres were calculated from regression data as the log_10_ reciprocal antibody dilution required for the 50% neutralization of 100 tissue culture infective units of virus (log_10_SN_50_/100 TCID_50_). The neutralizing antigenic relationship of viruses was given by the equation: the ratio r_1′_ = the neutralizing antibody titre against the heterologous virus/neutralizing antibody titre against the homologous virus. The significance of differences between “r_1_-values” obtained by the polyclonal antiserum was evaluated according to the criteria of Rweyemamu and Hingley [18]. According to these authors, *r*_1_-values of ≥0.3 are indicative of reasonable levels of cross-protection, whereas *r*_1_-values of <0.3 indicate very dissimilar strains and the need to acquire or develop another vaccine strain. All the tests were carried out at least twice, and the means of two tests were used in subsequent analysis.

### 2.3. RNA Extraction, Reverse Transcription (RT), Polymerase Chain Reaction (PCR), Nucleotide (nt) Sequencing and Analysis of the Sequence Data

To generate the sequence data of the capsid-coding region of the FMDVs, total RNA was extracted from cell culture-grown virus using a Qiagen Mini RNeasy extraction kit (Qiagen GmbH, Hilden, Germany) following the manufacturer’s guideline. RT-PCR to amplify the P1 region, sequencing, sequence analysis, assembling and alignment were performed as described previously [15]. Wherever necessary, new primers were designed (Table S2) and used to complete the sequence data for the full capsid-coding region of the FMDVs. The nucleotide sequences of the viruses were aligned using the CLUSTAL X multiple sequence alignment program [19], and the predicted amino acid (aa) sequences were translated using BioEdit 7.0.1 [20]. The alignments were used to construct distance matrices using the Kimura 2-parameter nucleotide substitution model as implemented in the program MEGA 6.0 [21].

### 2.4. Statistical Analysis

The statistical analysis was carried out using Minitab 17 software (http://www.minitab.com/en-us/products/minitab, accessed on 18 March 2021).

## 3. Result and Discussion

FMD is endemic in SEA and EA countries with the circulation of multiple serotypes and multiple genotypes within each serotype of the virus posing a threat to Australia and other FMD-free countries. Although vaccination is one of the most important control measures to prevent FMD outbreaks, the available vaccines may not be able to provide enough cross-protection against the FMDVs circulating in these countries due to the incursion of new lineages and sub-lineages as experienced in South Korea during 2010, a FMD-free country, when a new lineage of serotype O FMDV (Mya-98) spread to the country, highlighting the urgency of the following: (i) monitoring the circulating viruses in the region; and (ii) conducting regular vaccine-matching tests of circulating viruses with the existing vaccines in the antigenic reserve.

SEA countries, except Brunei Darussalam, Indonesia, Philippines and Singapore, are FMD-endemic and report regular outbreaks, of which more than 60% are caused by serotype O [8]. Currently, three topotypes of serotype O viruses (SEA, Cathay and ME-SA) are circulating in the region (Table 1), although historically SEA countries were home to SEA and Cathay topotype viruses. The O/ME-SA/PanAsia strain spread to SEA countries in early 2000, causing devastating economic consequences [12,13,14]. One decade later, in early 2010, another ME-SA strain (O/Ind-2001) from the Indian subcontinent spread to these countries [22]. The O/ME-SA/Ind-2001 viruses have now become established in the region and been detected in almost all FMD-endemic SEA countries, except Cambodia, Hong Kong and Thailand (Table 1), warranting studies to characterize these viruses both genetically and antigenically. In a previous publication, we conducted the antigenic and genetic characterization of serotype O FMDVs from the region, isolated over a six-year period (2007–2012), which revealed the existing serotype O vaccines to be a good match with the circulating viruses [12]. In this paper, we report the antigenic and genetic characteristics of serotype O FMDVs isolated over a six-year period (2013–2018) with the aim to study the antigenic nature of the viruses circulating in the region and/or detect the emergence of antigenic variants if any, as experienced previously in the Middle East necessitating the development of new vaccine strains for broad coverage [23].

### 3.1. Antigenic Characterization of Serotype O Viruses Circulating in SEA Countries

**Figure 1:**
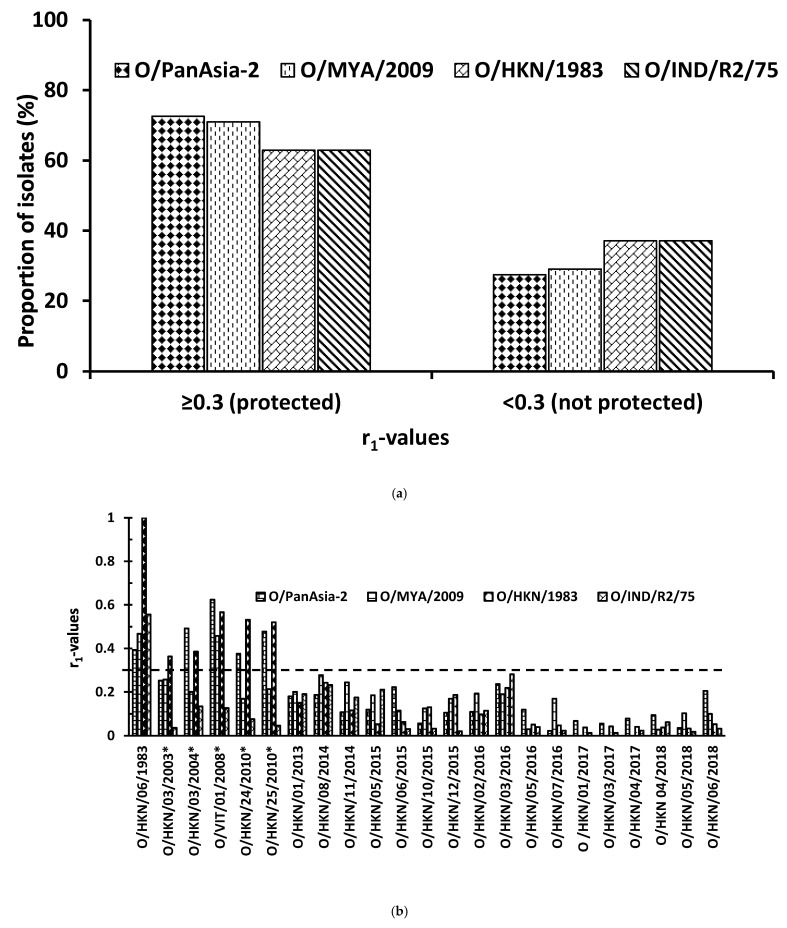
(**a**) Proportion of serotype O isolates (%) showing antigenic relationship (r_1_) values against four BVS. (**b**) Antigenic relationship (r_1_) values of serotype O/Cathay viruses against four BVS. The horizontal dotted line indicates the cut-off value of 0.3, above which the vaccine was considered to be a good match. The virus name with an asterisk at the end indicates the data taken from our previous study [12].

The cross-reactivities of four BVS against 62 serotype O viruses from nine different SEA countries were measured by 2D-VNTs in this study. Of these, the O/PanAsia-2 vaccine strain provided the maximum predicted protection (73%) followed by O/MYA/2009 (71%), while O/IND/R2/75 and O/HKN/1983 exhibited similar levels of in vitro protection (63%) (Figure 1a and Table 2a). The vaccine-matching data were analyzed further to study the predicted topotype-wise in vitro protection. The BVS from all the four *v*/*s* showed high cross-reactivity with the isolates from three topotypes/strains SEA/Mya-98, ME-SA/PanAsia and ME-SA/Ind-2001, whereas none of the isolates from Cathay topotype reacted with any of the BVS, not even the homologous O/HKN/1983 (Table 2b). O/PanAsia-2 was the best matched *v*/*s* for SEA/Mya-98 isolates, showing 100% in vitro protection, whereas O/HKN/1983 was the least matched *v*/*s* with an 88.46% match. Similarly, for ME-SA/PanAsia isolates, O/PanAsia-2 and O/MYA/2009 showed 100% in vitro protection, whereas O/HKN/1983 showed the least (76.92%) in vitro protection. The BVS of O/PanAsia-2, O/MYA/2009 and O/HKN/1983 showed 100% in vitro protection against O/ME-SA/Ind-2001 isolates, while the BVS of O/IND/R2/75 was comparatively less reactive, showing only a 66.67% in vitro match. This may not be an accurate reflection of the vaccine-matching result, as only six isolates belonging to the O/ME-SA/Ind-2001 strain were tested in this study. A country-wise analysis of the vaccine-matching data also showed similar results (Table 2a). Thus, it appears that the existing *v*/*s* are broadly cross-reactive and can provide protection against the circulating topotypes, except Cathay viruses, as demonstrated previously using O_1_/Campos *v*/*s* [24,25]. Several studies, mainly from South Korea, have reported the suitability of new putative strains for use in both pigs and cattle in their control programs [13,14,26].

### 3.2. Antigenic Drift in Serotype O/Cathay Viruses

The Cathay topotype viruses, characterized by a deletion within its 3A region [27], are pig-adapted strains and thought to emerge because of the movement of pigs or pig products across the border between China and Philippines in 1994 and that between China and Vietnam in 1997 [8]. It was detected in Hong Kong, Philippines, Vietnam, Thailand and Malaysia in 2005 [12,28] and also spread into Taiwan in 2009. Currently, this virus is mainly detected in China, Hong Kong, Taiwan and Vietnam (Table 1). The majority of FMD outbreaks in Hong Kong are caused by Cathay viruses, especially since 2013 (Table 1), although SEA/Mya-98 outbreaks were reported in 2010–2011 [8,12]. To the best of our knowledge, no in-depth studies have been carried out to characterize these viruses antigenically, although there are some reports on the genetic characterization of some isolates [29,30,31]. Therefore, a total of 17 Cathay viruses spanning a period of six years (2013–2018), with all from Hong Kong, were employed in this study. In addition, the data of five Cathay viruses employed in our previous antigen-matching study [12] were included in this study to obtain a broader picture of the antigenic evolution of these viruses. Interestingly, none of the BVS including the BVS raised against the O/Cathay *v*/*s* O/HKN/1983 matched with the viruses from Hong Kong from 2013 to 2018 (Figure 1b), whereas these BVS were a good match with viruses isolated from Hong Kong in 2003–2004 and 2010 and from Vietnam in 2008 (Figure 1b). Therefore, it warrants a close monitoring of these viruses and regular vaccine-matching work to ensure the *v*/*s* used in vaccination programs in the region is a good match. However, some recent studies have demonstrated the unreliability of only in vitro protection (r_1_-values) data to predict protection in several heterologous challenge experiments [24,25,32,33]. Therefore, although these *v*/*s* did not provide in vitro protection, it is possible that some protection could be observed in vivo, especially when high-antigen-payload vaccines are used. Indeed, a recent study involving a high-potency O/SKR/BOEUN/2017 vaccine (12.5 PD_50_) exhibited in vitro protection against Cathay virus [13,14]. In addition, a better in vivo correlation protection has been established, combining both virus neutralization test results and the T-cell responses [34], which was out of the scope of this study.

### 3.3. Genetic Characterization of Serotype O Viruses

The capsid-coding sequences of 54 serotype O FMDVs were generated in this study (Table S1; accession numbers: MZ851285–MZ851338). The capsid sequences of eight isolates (O/CAM/01/2013, O/CAM/02/2013, O/HKN/06/2018 O/TAI/27/2015, O/VIT/32/2013, O/VIT/39/2013, O/VIT/24/2014 and O/VIT/05/2015) used for antigenic characterization in this study could not be generated either because of problems in amplifying the capsid-encoding sequences or having ambiguities at more than eight nt positions that could not be resolved even after repeated attempts (data not shown). In addition, the capsid-coding sequences of five relevant isolates (four *v*/*s* used in this study and O/MYA/01/1998, the oldest virus of O/SEA/Mya-98 lineage—) were retrieved from GenBank and used for the genetic characterization of the SEA FMDVs, making it a total of 59 sequences. All the sequences were 2202 nucleotide long. Compared to O/HKN/1983, the oldest established Cathay topotype *v*/*s* of the region there was 11.98% (O/HKN/08/2014) to 16.89% (O/TAI/30/2015) variation at the nt level and 4.53% (O/HKN/06/2015 and O/HKN/05/2016) to 7.14% (O/LAO/01/2017 and O/TAI/30/2015) variation at the aa level. Similarly, compared to the oldest viral sequence of SEA/Mya-98 (i.e., O/MYA/01/98,) lineage employed in our analysis, the variation at the nt level was 7.01% (O/MYA/01/2015) to 17.99% (O/HKN/04/2018) and 2.2% (O/MAY/10/2014, O/VIT/21/2014) to 8.79% (O/HKN/04/2018) at the aa level.

**Figure 2:**
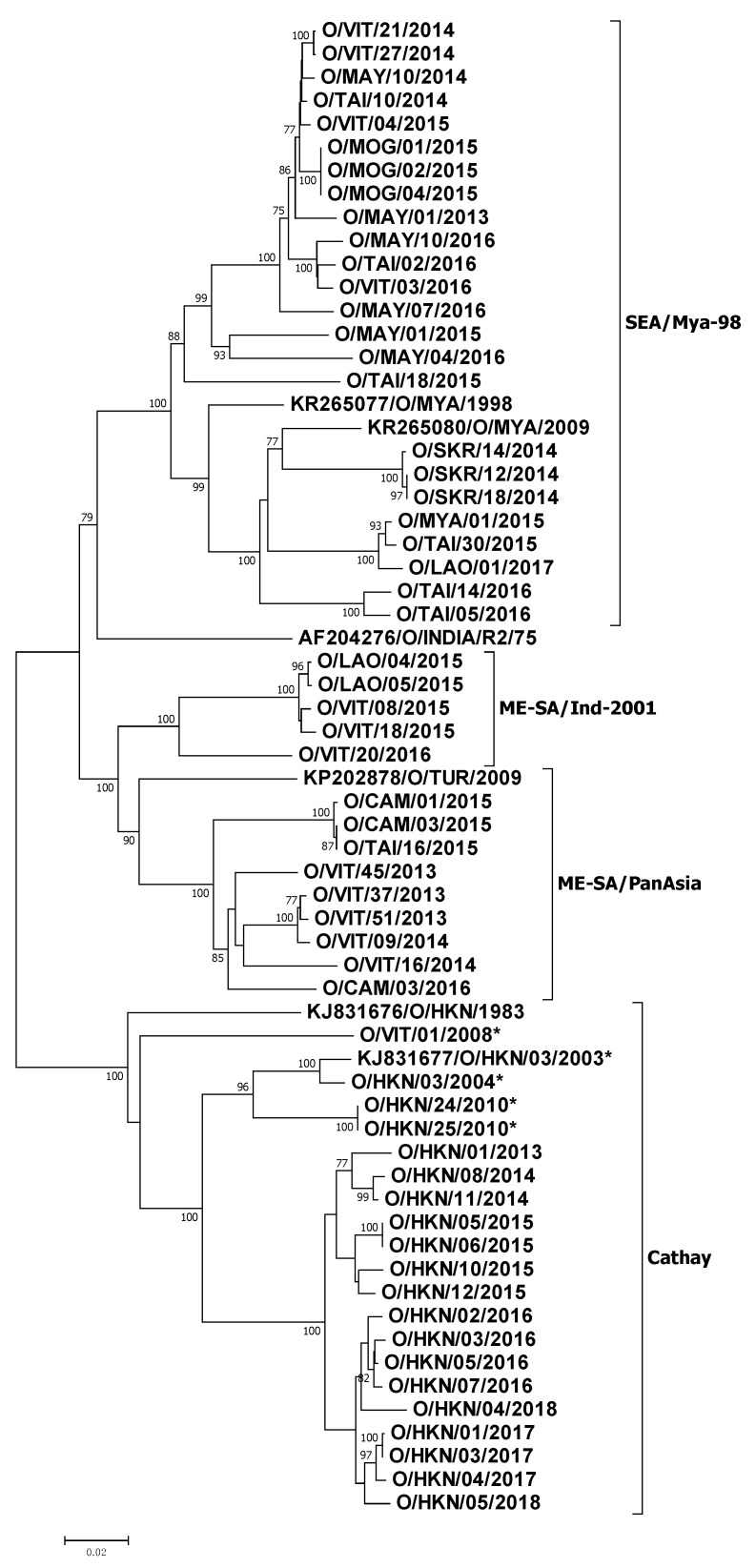
Neighbor-joining phylogenetic tree (P1) of the Southeast Asian serotype O viruses. The lineages/sub-lineages defined by WRLFMD on the basis of VP1 sequences are shown in the figure. The sequences with an asterisk at the end of their name are taken from our previous study [12] and included in the analysis to investigate the molecular basis of the antigenic drift in case of the Cathay topotype viruses. The GenBank accession numbers (MZ851285–MZ851338) of the sequences generated in this study are provided in Table S1. The tree was generated using MEGA 6.0 software [21].

A phylogenetic analysis of the capsid-coding sequences revealed the circulation of three topotypes (SEA, ME-SA and Cathay) of the serotype O viruses employed in this study (Figure 2). The ME-SA topotype viruses formed two distinct clusters representing viruses belonging to PanAsia and Ind-2001 lineages. All the Cathay topotype viruses from Hong Kong clustered together were separated from the O/HKN/1983 *v*/*s*, indicating these viruses have evolved away from the old strain. They also exhibited high levels of variation at the nt (11.98% to 13.57%) and aa (4.53% to 5.91%) levels, compared to the O/HKN/1983 *v*/*s*.

### 3.4. aa Variability of the Capsid of the Serotype O/Cathay Viruses

**Figure 3:**
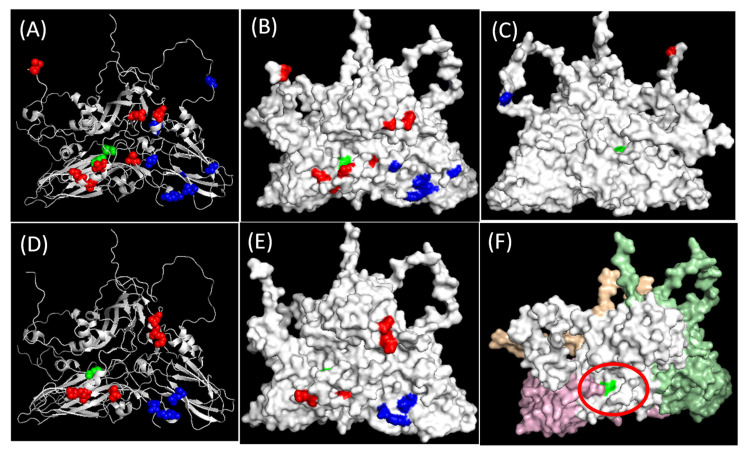
(**A**–**C**): Three-dimensional (3D) structure of the cartoon (**A**), external surface (**B**) and internal surface (**C**) of the O BFS-reduced protomer (1FOD, reduced) showing the aa variability of O SEA viruses. The residues in VP1, VP2 and VP3 are shown as red, green and blue spheres, respectively. (**D**–**F**). 3D structure of the cartoon (**D**), external surface (**E**) and internal surface (**F**) of the O BFS-reduced protomer (1FOD, reduced) showing aa residues, mainly responsible for the antigenic drift in serotype O/Cathay viruses as identified in this study, showing VP2-130 (shown in green) at the bottom of the pocket. The residues in VP1, VP2, VP3 and VP4 are shown in gray, pink, pale green and wheat, respectively. Red circle indicates the pocket, a possible antibody-binding site. The images were generated using PyMoL software (PyMOL Molecular Graphics System, Version 2.0, Schrodinger, LLC).

The variability of the capsid aa residues of the serotype O/Cathay viruses were analyzed further to understand the molecular basis of the antigenic drift. Similar to our earlier reports, VP4 was found to be highly conserved, whereas VP1 was least conserved (data not shown) [15]. The analysis of the aa sequences identified a total of 11 residues (two in VP2, four in VP3 and five in VP1) with variability scores greater than 0.6 (Table 3a). Of these, four (one each in VP2 and VP3 and two in VP1) had very high scores (above 0.9; Table 3a). These residues could be of antigenic significance, as they are either part of neutralizing antigenic sites, for example VP1 47 (antigenic site 3), or located in an adjacent area that are known to strongly influence the binding of neutralizing mabs [35,36,37]. All these residues were found to be surface-exposed, except one in VP2 (VP2-163) and two residues in VP3 (VP3-36 and VP3-99) (Figure 3A–C).

### 3.5. Correlating Genotype to Antigenic Phenotype of Serotype O/Cathay Viruses

The in vitro testing of the viruses belonging to O/Cathay topotype from Hong Kong (2013–2018) with all the four BVS used in this study generated low *r*_1_-values, indicating lower expected protection. Of these BVS, O/PanAsia-2 and O/HKN/1983 were a good match with the viruses up to the year 2010, although very few viruses were included in the study [12]. The capsid aa sequences of these Cathay viruses, including the sequences of five isolates previously reported [12] or retrieved from GenBank, were analyzed further to understand the molecular basis of the antigenic drift of these viruses. As none of these viruses cross-reacted with the BVS of any of the *v*/*s* used in this study, we specifically looked for aa residues in the field isolates, which were different from those of all the four *v*/*s* and also from the sequence of the isolates of 2010 and before. A total of nine aa residues (five in VP1, one in VP2 and three in VP3) were identified (Table 3b and Figure 3D–F)) that could explain the antigenic drift of these viruses. All these nine aa residues were found to be on the surface (Figure 3E). Of these, one residue (VP2-130) is noteworthy, as it was located at the bottom of the pocket present at the junction between VP1 and VP2 (Figure 3F). This pocket was formed by residues VP2 130-34, known to influence the binding of antigenic site 2 mAbs [36,38] and the G-H loop residues of VP1, which are known to contain the integrin receptor and form neutralizing antigenic site 1 in case of serotypes O, A, Asia 1 and SAT 1-2 [35,38,39,40,41,42,43,44,45,46], and could be biologically important. In case of rhinoviruses, the canyon regions were shown to contain the binding sites for the cellular receptor [47]. In hepatitis B virus (HBV), substitutions in the hydrophobic pocket of the core protein resulted in a significant conformational alternation of the capsid [48]. This residue (VP2 130) also had a very high variability score with four alternate aa residues (Table 3A). Of the remaining eight residues, VP1 96 had a substitution from a polar (T) to a non-polar (A) aa residue, whereas two positions each had changes to a negatively (VP3-131 and -134) or a positively charged (VP3-76 and VP1-149) aa residue. Of the residues identified in VP1, VP1-141 was within antigenic site 1, while VP1-149 formed antigenic site 5 in serotype O viruses [35,42]. In addition, the antigenic significance of both VP1-149 and -150 have been reported in serotype A viruses [37]. Studying the antigenic and genetic characteristics of the serotype O/Cathay viruses between 2010 and 2013 could elucidate the antigenic drift of these viruses better, which was not possible within the scope of this study. Moreover, as not all the FMD outbreaks are reported or investigated gaps such as the non-availability of viruses in some years (a serotype O/Cathay outbreak was reported in Hong Kong in 2011, but none was reported in 2012 [12]), the lack of funding to carry out systematic studies to understand the epidemiology and evolution of these viruses including antigenic characteristics could be serious impediments to fully understand the molecular basis of the observed antigenic drift.

## 4. Conclusions

In conclusion, the serotype O *v*/*s* used in this study are a good match with the circulating field isolates belonging to the ME-SA and SEA strains, with O/PanAsia-2 BVS being the most reactive vaccine followed by O/MYA/2009. However, none of the *v*/*s* could protect the O/Cathay viruses from Hong Kong, indicating an antigenic drift in case of these viruses. The analysis of the capsid sequences revealed several aa substitutions in neutralizing antigenic sites, which could explain the observed antigenic drift. Targeted mutagenesis studies involving a serotype O infectious clone could confirmed these observations. It is evident from the result of this study that the serotype O *v*/*s* are broadly cross-reactive and, could be used as a vaccine to control the outbreaks caused by SEA/Mya-98, ME-SA/Ind-2001 and ME-SA/PanAsia viruses in the region. In contrast, the *v*/*s* used in this study appear to be not a good match with the O/Cathay viruses, warranting a close monitoring of these viruses and a regular vaccine-matching (both in vitro and in vivo) study to evaluate the suitability of the existing *v*/*s* for use in FMD control programs; and if required, a new *v*/*s* with high immunogenicity and a broad antigenic spectrum with FMDVs representing topotypes/lineages from SEA countries needs to be developed in a timely manner to prevent future outbreaks.

## Figures and Tables

**Table 1:** List of serotype O foot and mouth disease virus (FMDV) strains detected in various Southeast Asia (SEA), East Asia (EA) and neighboring countries during 2013–2020.

Country	Cathay	SEA/Mya-98	Middle East-South Asia (ME-SA)/PanAsia	ME-SA/Ind-2001
**Cambodia**	-	-	2013, 2015–2016, 2018	-
**China**	2013, 2016, 2018	2013, 2018	2018	2017–2018
**Hong Kong**	2013–2019	-	-	-
**Lao PDR**	-	2013, 2016–2017	2018	2015, 2020
**Malaysia**	-	2013–2016	-	2016–2018
**Mongolia**	-	2015, 2018	2014, 2017–2018	2015, 2017–2018
**Myanmar**	-	2013, 2015, 2017	-	2016–2018
**Russia**	-	2019–2020	2014, 2017–2018	2016, 2019
**South Korea**	-	2014–2016	-	2017, 2019
**Taiwan**	2013	-	-	-
**Thailand**	-	2013–2018	2015, 2017–2018	2016–2018, 2020
**Vietnam**	2016–2018	2014–2019	2013–2014, 2017–2019	2015–2017, 2019–2020

**Table 2:** (**a**) List of SEA and EA countries showing % in vitro protection by each vaccine strain. (**b**). List showing the number of FMDV isolates from each lineage with positive cross-reactivity (r_1_-values ≥ 0.3) with bovine post-vaccinal sera (BVS).

(a)					
Country/Vaccine	No. of Virus Tested	O/PanAsia-2	O/MYA/2009	O/HKN/1983	O/IND/R2/75
**Cambodia**	5	100	100	80	60
**Hong Kong**	17	0	0	0	0
**Lao**	3	100	100	100	100
**Malyasia**	6	100	100	100	100
**Mongolia**	3	100	100	100	100
**Myanmar**	1	100	100	100	100
**South Korea**	3	100	100	0	100
**Thailand**	8	100	87.5	100	75
**Vietnam**	16	100	100	87.50	87.50
**(b)**					
**Vaccine/Lineage**	**Cathay**	**Mya-98**	**PanAsia**	**Ind-2001**	**Total**
**No. of virus tested**	17	26	13	6	62
**O/PanAsia-2**	0	26	13	6	45
**O/MYA/2009**	0	25	13	6	44
**O/HKN/1983**	0	23	10	6	39
**O/IND/R2/75**	0	24	11	4	39

**Table 3:** (**a**). Capsid positions with high variability scores (>0.6) and corresponding aa changes. The residues with scores above 0.9 are shaded gray. aa: amino acid. (**b**) Capsid sequence changes observed in Cathay serotype O viruses used in this study.

(a)													
Position							aa Changes						
VP2-130							C/V/A/D						
VP2-163							V/L						
VP3-36							L/M						
VP3-59							G/Y/D						
VP3-99							T/A						
VP3-174							V/I/T						
VP1-47							Q/K						
VP1-134							Q/S/N						
VP1-148							L/F						
VP1-150							V/E/Q						
VP1-155							A/V						
(**b**)													
**Serial. No.**	**Virus Name**	**VP2-130**	**VP3-76**	**VP3-131**	**VP3-134**	**VP3-174**	**VP1-96**	**VP1-99**	**VP1-141**	**VP1-148**	**VP1-149**	**VP1-150**	**VP1-155**
1	O/IND/R2/75	C	Q	E	K	T	N	D	V	L	Q	V	A
2	O/MYA/2009	C	Q	E	K	A	A	D	L	L	Q	V	A
3	O/PanAsia-2	C	Q	E	K	T	T	D	T	L	Q	V	A
4	O/HKN/06/1983	C	Q	E	K	V	T	D	V	L	Q	V	A
5	O/HKN/03/2003 *	C	Q	E	K	V	T	D	V-T	L	Q	V	A
6	O/HKN/03/2004 *	C	Q	E	K	V	T	D	V-T	L	Q	V	A
7	O/VIT/01/2008 *	C	Q	E	K	V	T	D	V-T	L	Q	V	A
8	O/HKN/24/2010 *	C	Q	E	K	V	T	D	V-A	L	Q-H	V	A
9	O/HKN/25/2010 *	C	Q	E	K	V	T	D	V-A	L	Q-H	V	A
10	O/HKN/01/2013	C-V	Q-R	E-D	K-E	V	T-A	D-N	V-N	L-F	Q-R	V-E	A-V
11	O/HKN/08/2014	C-V	Q-R	E-D	K-E	V-I	T-A	D-N	V-N	L-F	Q-R	V-E	A-V
12	O/HKN/11/2014	C-V	Q-R	E-D	K-E	V-I	T-A	D-N	V-N	L-F	Q-R	V-E	A-V
13	O/HKN/05/2015	C-V	Q-R	E-D	K-E	V-I	T-A	D-N	V-N	L	Q-R	V-Q	A
14	O/HKN/06/2015	C-V	Q-R	E-D	K-E	V-I	T-A	D-N	V-N	L	Q-R	V-Q	A
15	O/HKN/10/2015	C-A	Q-R	E-D	K-E	V	T-A	D-N	V-N	L	Q-R	V-Q	A
16	O/HKN/12/2015	C-V	Q-R	E-D	K-E	V	T-A	D-N	V-N	L	Q-R	V-Q	A
17	O/HKN/02/2016	C-D	Q-R	E-D	K-E	V-I	T-A	D-N	V-N	L	Q-R	V-Q	A
18	O/HKN/03/2016	C-V	Q-R	E-D	K-E	V-I	T-A	D-N	V-N	L	Q-R	V-Q	A
19	O/HKN/05/2016	C-D	Q-R	E-D	K-E	V-I	T-A	D-N	V-N	L	Q-R	V-Q	A
20	O/HKN/07/2016	C-V	Q-R	E-D	K-E	V-I	T-A	D-N	V-N	L	Q-R	V-Q	A
21	O /HKN/01/2017	C-V	Q-R	E-D	K-E	V-I	T-A	D-N	V-N	L	Q-R	V-Q	A
22	O/HKN/03/2017	C-V	Q-R	E-D	K-E	V-I	T-A	D-N	V-N	L	Q-R	V-Q	A
23	O/HKN/04/2017	C-V	Q-R	E-D	K-E	V-I	T-A	D-N	V-N	L	Q-R	V-Q	A
24	O/HKN 04/2018	C-V	Q-R	E-D	K-E	V-I	T-A	D-N	V-N	L	Q-R	V-Q	A
25	O/HKN/05/2018	C-V	Q-R	E-D	K-E	V	T-A	D-N	V-N	L	Q-R	V-Q	A
26	O/HKN/06/2018 ^#^	C-V	Q-R	E-D	K-E	V	T-A	D-N	V-N	L	Q-R	V-Q	A

The virus sequences (*n* = 16) were generated in this study, and the sequences with an * at the end of their name (*n* = 10) were either generated in our previous study [12] or extracted from GenBank. # at the end of the virus name indicates the isolate which was partially sequenced. The sequence changes that appear to be mainly responsible for the antigenic drift of the recent Cathay topotype viruses are shaded gray.

## Data Availability

The sequence datasets generated during this research have been submitted to NCBI, and accession numbers have been awaited.

## Supplementary Materials

The following are available online at www.mdpi.com/article/10.3390/v13091886/s1, Table S1: List of serotype O FMDVs used in this study. Table S2: List of primers used for sequencing.
