# Supplementary material for: Molecular Basis of Antigenic Drift in Serotype O Foot-and-Mouth Disease Viruses (2013–2018) from Southeast Asia
Source: Viruses. 2021 Sep 21;13(9):1886. doi: 10.3390/v13091886 (PMC8473337; doi:10.3390/v13091886)
Supplement: Supplementary file 1 [file viruses-13-01886-s001.zip › viruses-1336665-supplementary.pdf]

viruses

Supplementary Materials

Molecular Basis of Antigenic Drift in Serotype O Foot-and-Mouth Disease Viruses (2013–2018) from Southeast Asia

Table S1. List of serotype O FMD viruses used in this study.

SL NO	Virus Name	Country	Region	Date Collected	Host	Topo-type	Strain	Acces-sion No
1	O/HKN/1983*	Hong Kong	Hong Kong	1983	Pig	CA-THAY		KJ831676
2	O/IND/R2/75*	India	India	1975	NK	ME-SA	Branch B	AF204276
3	O/MYA/2009*	Myanmar	Magway, Myanmar	29/10/2009	Cattle	SEA	Mya-98	KR265080
4	O/PanA-sia-2*	Turkey	Turkey	01/01/2009	Cattle	ME-SA	PanA-sia	KP202878
5	O/CAM/01/2013	Cambodia	Kampong Speu Province	19/09/2013	Cattle	ME-SA	PanA-sia	ND
6	O/CAM/02/2013	Cambodia	Svay Rieng Province	19/09/2013	Cattle	ME-SA	PanA-sia	ND
7	O/CAM/01/2015	Cambodia	Bakan	14/09/2015	Pig	ME-SA	PanA-sia	MZ851285
8	O/CAM/03/2015	Cambodia	Bakan	14/09/2015	Pig	ME-SA	PanA-sia	MZ851286
9	O/CAM/03/2016	Cambodia	Domreyslab, Tree commune, Kandalsteung, Kandal	02/12/2016	Pig	ME-SA	PanA-sia	MZ851287
10	O/HKN/01/2013	Hong Kong	NK	02/04/2013	Pig	CA-THAY	N/A	MZ851288
11	O/HKN/08/2014	Hong Kong	NK	24/02/2014	Pig	CA-THAY	N/A	MZ851289
12	O/HKN/11/2014	Hong Kong	NK	06/03/2014	Pig	CA-THAY	N/A	MZ851290
13	O/HKN/05/2015	Hong Kong	Sai Kung, New Territories	18/09/2015	Porcine	CA-THAY	N/A	MZ851291
14	O/HKN/06/2015	Hong Kong	Sai Kung, New Territories	18/09/2015	Porcine	CA-THAY	N/A	MZ851292
15	O/HKN/10/2015	Hong Kong	NK	07/11/2015	Porcine	CA-THAY	N/A	MZ851293
16	O/HKN/12/2015	Hong Kong	Shueng Shui	02/12/2015	Pig	CA-THAY	N/A	MZ851294
17	O/HKN/02/2016	Hong Kong	Sai Kung, New Territories	06/08/2016	Porcine	CA-THAY	N/A	MZ851295
18	O/HKN/03/2016	Hong Kong	Sai Kung, New Territories	06/08/2016	Pig	CA-THAY	N/A	MZ851296
19	O/HKN/05/2016	Hong Kong	Sai Kung, New Territories	06/08/2016	Porcine	CA-THAY	N/A	MZ851297
20	O/HKN/07/2016	Hong Kong	Sai Kung, New Territories	06/08/2016	Pig	CA-THAY	N/A	MZ851298
21	O/HKN/01/2017	Hong Kong	Sheung Shui, New Territorie	11/09/2017	Porcine	CA-THAY	N/A	MZ851299
22	O/HKN/03/2017	Hong Kong	Sheung Shui, New Territories	11/09/2017	Porcine	CA-THAY	N/A	MZ851300

23	O/HKN/04/2017	Hong Kong	Sheung Shui, New Territories	14/09/2017	Porcine	CA-THAY	N/A	MZ851301
24	O/HKN/04/2018	Hong Kong	San Tin, Yuen Long, New Territories	28/02/2018	Porcine	CA-THAY	N/A	MZ851302
25	O/HKN/05/2018	Hong Kong	San Tin, Yuen Long, New Territories	07/03/2018	Porcine	CA-THAY	N/A	MZ851303
26	O/HKN/06/2018	Hong Kong	San Tin, Yuen Long, New Territories	07/03/2018	Porcine	CA-THAY	N/A	ND
27	O/LAO/04/2015	Laos	Naxaythong, Vientiane	10/06/2015	NK	ME-SA	Ind-2001d	MZ851304
28	O/LAO/05/2015	Laos	Naxaythong, Vientiane	10/06/2015	NK	ME-SA	Ind-2001d	MZ851305
29	O/LAO/01/2017	Laos	Vangpho, Parknguem, Vientiane City	30/01/2017	Cattle	SEA	Mya-98	MZ851306
30	O/MAY/01/2013	Malaysia	Kulaijaya, Johor	06/01/2013	Cattle	SEA	Mya-98	MZ851307
31	O/MAY/10/2014	Malaysia	Bachok, Perupok, Kelantan	01/07/2014	cattle	SEA	Mya-98	MZ851308
32	O/MAY/01/2015	Malaysia	Kuala Berang, Terengganu	26/03/2015	cattle	SEA	Mya-98	MZ851309
33	O/MAY/04/2016	Malaysia	Jasin, Selandar, Melaka	14/03/2016	cattle	SEA	Mya-98	MZ851310
34	O/MAY/07/2016	Malaysia	Hilir Perak, Hutan Melintang, Perak	02/06/2016	cattle	SEA	Mya-98	MZ851311
35	O/MAY/10/2016	Malaysia	Melaka Tengah, Ayer Molek, Melaka	08/08/2016	cattle	SEA	Mya-98	MZ851312
36	O/MOG/01/2015	Mongolia	NK	28/02/2015	Cattle	SEA	Mya-98	MZ851313
37	O/MOG/02/2015	Mongolia	NK	28/02/2015	Cattle	SEA	Mya-98	MZ851314
38	O/MOG/04/2015	Mongolia	NK	28/02/2015	Cattle	SEA	Mya-98	MZ851315
39	O/MYA/01/2015	Myanmar	Mahlaing, Mandalay	29/10/2015	Bovine	SEA	Mya-98	MZ851316
40	O/SKR/12/2014	Republic of Korea	NK	03/12/2014	Pig	SEA	Mya-98	MZ851317
41	O/SKR/14/2014	Republic of Korea	NK	03/12/2014	Pig	SEA	Mya-98	MZ851318
42	O/SKR/18/2014	Republic of Korea	NK	03/12/2014	Pig	SEA	Mya-98	MZ851319
43	O/TAI/10/2014	Thailand	Lai Nan, Wiang Sa, Nan, Thailand	10/03/2014	Cattle	SEA	Mya-98	MZ851320
44	O/TAI/16/2015	Thailand	Lumpayaklang, Mouklek, Saraburi, Thailand	20/05/2015	Cattle	ME-SA	PanAsia	MZ851321
45	O/TAI/18/2015	Thailand	Banlao, Mayjai, Payao	28/07/2015	Water Buffalo	SEA	Mya-98	MZ851322
46	O/TAI/27/2015	Thailand	Kangkajan, Kangkajan, Petchburi	28/09/2015	Cattle	SEA	Mya-98	ND
47	O/TAI/30/2015	Thailand	Chachang, Sunkumpang, Chaing Mai	13/11/2015	Cattle	SEA	Mya-98	MZ851323
48	O/TAI/02/2016	Thailand	Huanadum, Kanoun, Khonkean	11/01/2016	Cattle	SEA	Mya-98	MZ851324
49	O/TAI/05/2016	Thailand	Nong Or, Ban Pong, Ratchburi	26/01/2016	Pig	SEA	Mya-98	MZ851325

50	O/TAI/14 /2016	Thailand	Wokeaw, Hangchath, Lumpang	03/03/2016	Cattle	SEA	Mya-98	MZ851326
51	O/VIT/32 /2013	Vietnam	Son La	30/05/2013	Cattle	ME-SA	PanA-sia	ND
52	O/VIT/37 /2013	Vietnam	Bac Ninh	13/08/2013	Cattle	ME-SA	PanA-sia	MZ851327
53	O/VIT/39 /2013	Vietnam	Bac Can,	24/08/2013	Cattle	ME-SA	PanA-sia	ND
54	O/VIT/45 /2013	Vietnam	Quang Tri	23/10/2013	Water Buffalo	ME-SA	PanA-sia	MZ851328
55	O/VIT/51 /2013	Vietnam	Lam Dong	30/10/2013	Cattle	ME-SA	PanA-sia	MZ851329
56	O/VIT/09 /2014	Vietnam	Son La,	24/04/2014	Cattle	ME-SA	PanA-sia	MZ851330
57	O/VIT/16 /2014	Vietnam	Tien Giang	30/04/2014	Pig	ME-SA	PanA-sia	MZ851331
58	O/VIT/21 /2014	Vietnam	Quang Nam	20/06/2014	Cattle	SEA	Mya-98	MZ851332
59	O/VIT/24 /2014	Vietnam	Long An	10/07/2014	Pig	ME-SA	PanA-sia	ND
60	O/VIT/27 /2014	Vietnam	Kon Tum	17/07/2014	Cattle	SEA	Mya-98	MZ851333
61	O/VIT/04 /2015	Vietnam	Long An	27/04/2015	Cattle	SEA	Mya-98	MZ851334
62	O/VIT/05 /2015	Vietnam	Lao Cai	10/07/2015	Cattle	ME-SA	Ind-2001d	ND
63	O/VIT/08 /2015	Vietnam	Lao Cai	10/07/2015	Cattle	ME-SA	Ind-2001d	MZ851335
64	O/VIT/18 /2015	Vietnam	Yen Bai	02/12/2015	Cattle	ME-SA	Ind-2001d	MZ851336
65	O/VIT/03 /2016	Vietnam	Quang Nam	29/02/2016	Cattle	SEA	Mya-98	MZ851337
66	O/VIT/20 /2016	Vietnam	Son La	23/09/2016	Cattle	ME-SA	Ind-2001d	MZ851338

ME-SA: Middle East-South Asia; SEA: Southeast Asia; NK: not known; ND: not done. The virus name with an asterisk (*) at the end (n = 4) were retrieved from GenBank and 54 capsid sequences were generated in this study.

Table S2. List of forward and reverse primers used to sequence the capsid coding region of serotype O FMD viruses used in this study.

SL. No.	Name	Sequence (5'--------3')
1	L463F	ACCTCCRACGGGTGGTACGC
2	L490F	GAYGAGGAITTYTACCCITGGAC
3	OIA67F	AACAAYTACTACATGCARCA
4	OIB253F	ACIGACCACAARGGYGTCTA
5	OIB439F	TTCATYAACCCIMGIACGAACATGAC
6	OIB454F	ACGAACATGACRGCICACATC
7	O1B535F	CTIGTGGTYATGGTYGTRGC
8	O1C244F	GCAGCAAAACACATGTCAAACACCTT
9	01C272F	TBGCRGGNCTYGCCCAGTACTAC
10	OIC283F	GCCCAGTACTACACACAGTACAG
11	OIC499F	TACGCGTACACCGCGTC
12	OIC564F	AATTACACATGGCAAGGCCGACACGG
13	OIC583F	GACGGYGAYGCICTGGTCGT
14	O1D293F	TGGAYAACACCACYAAYCCAAC
15	OID296F	ACAACACCACCAACCCAAC

16	O1B40R	CCGTTRCGRGTRGTGAGGATGCGGTC
17	OIB190R	GTGACCCARTCRAAIARRTGGGT
18	OIB200R	CGAGGAGCTRGTRACCCAGTC
19	OIB253R	GCCGTAGACRCCYTTCTGGTCAGT
20	OIB439R	GTCATGTTCGTICKIGGGTTRATGAA
21	OIB529R	ACRACCATRACCACRAGGGTCCA
22	OIC286R	GTGCCRCTGTACTGKGTGTAGTACTG
23	OIC329R	TTGAACACTTTCCCGTA
24	OIC361R	GGI GGG GCR TAI GCA AYC ATG TA
25	OIC386R	GTGTTTGACATGTGCTTTG
26	O1D95R	ATGAACGAGACRTCYGTGTG
27	OID125R	TGTCACYTTCACRAAYCT
28	OID475R	ATIGCICCRWARTTRAARGAIGTIGGCAG
29	OID628R	GTTGGGTTGGTGGTGTTGT
30	EUR2B52R	GACATGTCCTCCTGCATCTGGTTGAT
31	NK72	GAAGGGCCCAGGGTTGGACTC
